# Utilization of, satisfaction toward, and challenges for Internet-based healthcare services provided by primary health institutions: Evidence from China

**DOI:** 10.3389/fpubh.2022.1100634

**Published:** 2023-01-19

**Authors:** Shuo Wang, Xin Wang, Yuyin Zhou, Junfang Xu

**Affiliations:** ^1^Center for Health Policy Studies, School of Public Health, Zhejiang University School of Medicine, Hangzhou, China; ^2^Department of Pharmacy, Second Affiliated Hospital, Zhejiang University School of Medicine, Hangzhou, China; ^3^School of Public Health, Health Development Research Center, Sun Yat-sen University, Guangzhou, China

**Keywords:** utilization, satisfaction, challenges, Internet-based healthcare, primary health institutions

## Abstract

**Background:**

The rapid development of “Internet plus healthcare” in China has provided new ways for the innovative development of primary healthcare. In addition, a series of favorable policies have been issued to promote Internet-based healthcare services in primary health institutions.

**Objective:**

The aim of this study was to describe the utilization of, satisfaction toward, and challenges faced by Internet-based healthcare services provided by primary health institutions in China.

**Methods:**

A self-designed structured questionnaire was employed to collect related data in January 2022 through Credamo. The questionnaire mainly included sociodemographic characteristics, health-related information, utilization of, satisfaction toward, and challenges faced by Internet-based healthcare services provided by primary health institutions. Descriptive analysis was used to describe the sociodemographic characteristics, utilization, satisfaction, and challenges by subgroups. The Wilcoxon rank-sum test was carried out to examine the differences in satisfaction with Internet-based healthcare services between participants who ever received these services and those who did not. A multiple logistic regression model was also used to examine the factors influencing the utilization of Internet-based healthcare services provided by primary health institutions.

**Results:**

A total of 10,600 residents were included in the final analysis, of whom 5,754 (54.3%) were women. Overall, 51.3% (5,434) of the total participants ever used Internet-based healthcare services provided by primary health institutions. Among those who used Internet-based healthcare services, the most widely used services were procedure-related consultation services (63.7%). The satisfaction among those who ever used it was significantly higher than that among those who did not (84.7 vs. 45.4%; *p*-value < 0.001). One of the biggest challenges (69.3%) expressed by the residents was that it was difficult for the elderly to use Internet-based services, followed by community doctors with low capacity of providing primary care online (49.0%) and residents were worried about the information security and privacy protection (48.5%). Younger people, people with lower education levels, and people with chronic diseases were significantly more likely to use Internet-based healthcare services provided by primary health institutions (*P* < 0.05).

**Conclusion:**

Among 10,600 residents surveyed in China in 2022, more than half of the people used Internet-based healthcare services provided by primary health institutions, and most of them were satisfied, although subgroups significant differences existed. The most common use was procedure-related (e.g., online registration and result query), and several challenges of using Internet-based healthcare services exist (e.g., information safety and usage among elderly people). Therefore, it is important to further improve Internet-based primary healthcare services based on the population perception of achieving healthy China in 2030.

## Introduction

With the rapid development of the Internet and information technologies, more than one billion people were reported to have access to the Internet in 2021, and about 73% of people have a smartphone or smart device to search on the Internet in China (1). Correspondingly, health-related services based on the Internet, commonly called “Internet plus healthcare,” were developed gradually, which was a novel application of the Internet in the healthcare industry that includes health education and intervention, medical information records and queries, electronic prescriptions, online and remote consultations, and various remote forms of preventive, treatment, and rehabilitation-oriented health services (2, 3). In reality, the popularization of Internet-based healthcare services could improve access to healthcare or high-quality health services with lower medical expenditure, which can be a supplement to medical resources, especially for people in rural or remote areas (4–6).

As a new model, Internet plus healthcare also has the potential to change the nature of some service delivery, especially primary healthcare with its essentially “generalist” characteristic. Moreover, the traditional service delivery in primary health institutions was challenged by limited general practitioners, weak service capacity, and declining utilization influenced by the COVID-19 pandemic. Under this background, new ways emerged for promoting the provision of primary healthcare, such as online chronic disease management, Internet-based preventive healthcare, and health education provision. A series of favorable policies were also issued by the national government for primary healthcare recently in China. In 2018, the State Council proposed to use Internet technology to improve the capacity and efficiency of primary health services and promote the construction of an orderly hierarchical diagnosis and treatment system (7). In 2020, the Ministry of Industry and Information Technology and the National Health Commission jointly set the goal of connecting more than 98% of primary health institutions to the Internet by the end of 2022 (8). The Internet-based healthcare services provided by primary health institutions also played a positive role in meeting the medical needs of patients and alleviating the pressure of higher level hospitals, especially in remote and rural areas (9). Moreover, during the COVID-19 epidemic period, in terms of regular medical care, at least 940,182 doctors provided online care, and 13.7 million provided remote consultations (10). Based on our investigations with 496 staff from 93 primary health institutions in 2022, 73.1% of health education sessions on infectious diseases were carried out based on the Internet, with 63.8% for township hospitals and 87.5% for primary health centers. In addition, evidence from developed countries has shown that providing prevention, public health, and rehabilitation services online was highly feasible and cost-effective (10–13), which were the main services of primary health institutions. Previous studies discussed Internet use mostly at higher level hospitals (e.g., tertiary hospitals), a few studies focused on primary health institutions, and little is known about the utilization of, satisfaction toward, and challenges faced by Internet-based health services provided by primary health institutions in China. The few studies on the use of Internet-based healthcare in China have shown that people who use the Internet for health-related purposes are more likely to be young and female, with high levels of income and education, or have poorer health status (5). Some researchers noted that online health services help foster positive interactions between patients and physicians and improve patients' trust in their physicians. Therefore, the aims of our study were to describe the use and satisfaction toward the future of health-related Internet use, explore the challenges that Internet-based services faced at primary health institutions, and provide evidence for the promotion of primary health institution development through the Internet.

## Methods

### Data collection

A self-designed structured questionnaire was employed to collect data in January 2022 through Credamo, which is a professional online survey platform and works similarly to MTurk in providing research services in China (14). All participants older than 18 years were welcome and included in our study. Theoretically, at least 605 participants were required based on 5–10 times of entries of the questionnaire considering 10% of invalid questionnaires.

The questionnaire was designed based on previous studies and qualitative investigation with eight providers of primary health institutions from Zhejiang province based on purposive sampling. Based on previous studies and qualitative evidence from providers regarding the common and expected Internet medical services, challenges or shortcomings for Internet medical services in primary health institutions, the questions on the use and challenges of Internet-based healthcare services provided by primary health institutions were designed. The type of Internet-based healthcare services provided by primary health institutions included online registration/appointments, online health results query, electronic prescription, online public health education, remote consultation, online hypertension health management, online diabetes health management, online joint consultation, and electronic signature. The challenges of Internet-based healthcare services provided by primary health institutions included the following contents: It is difficult for the elderly to use Internet-related services, community doctors with low capacity of providing primary care online, residents are worried about the information security and privacy protection, community rental personnel flow is large, the function of intelligent service needs to be upgraded, and information operation fund is scarce.

In addition to these, the questionnaire also included the following parts: basic characteristics about the residents (e.g., gender, age, monthly income, occupation, educational level, marital status, household registration, medical insurance, and whether contracted with a general practice); health-related information (e.g., self-rated health score, whether diagnosed with chronic diseases, and the distance to the nearest health institutions); and satisfaction with Internet-based healthcare services provided by primary health institutions.

All participants were asked to rate their satisfaction with the Internet-based healthcare services using very satisfied, satisfied, tolerable, and not satisfied. In addition, we examined the reliability and validity of the questionnaires using Cronbach's α coefficient (α = 0.877), which showed that the questionnaire exhibited high reliability and validity. Finally, a total of 10,850 people participated in our survey and 10,600 participants were incorporated in the data analysis after deleting the invalid cases.

### Data analysis

Descriptive analysis was used to describe the demographic characteristics (e.g., gender, age, monthly income, occupation, educational level, marital status, household registration, and medical insurance) of participants. Considering the satisfaction with Internet-based healthcare services is ranked data, the Wilcoxon rank-sum test was carried out to examine the differences in satisfaction with Internet-based healthcare services between participants who ever used these services and those who did not. Moreover, the association between respondents' characteristics (e.g., gender, age, and household registration) and their utilization of Internet-based healthcare services provided by primary health institutions was assessed using Pearson's chi-square test. Multivariate logistic regression was used to analyze the factors that may be associated with the utilization of Internet-based healthcare services provided by primary health institutions, and odds ratios (OR) were reported. SAS 9.4 software was applied for the statistical analysis of data with *P* < 0.05 as the cutoff for statistical significance.

## Results

Table 1 shows the characteristics of participants in our study. Approximately half of all participants were female (*N* = 5,754, 54.3%), 57.7% (*N* = 6,119) of participants were <30 years old, and 41.5% (*N* = 4,402) were 30–60 years old. More than 80% of participants had an education level higher than senior high school, and most of the participants (*N* = 9,268, 87.5%) had a monthly income of <10 thousand RMB ($1,487). Among participants, 36.4% (*N* = 3,862) of them had a new rural cooperative medical system (NRCMS), 39.7% (*N* = 4,212) had a basic medical insurance system for urban residents (UCBMI), 36.2% (*N* = 3,842) had a basic medical insurance system for the urban employee (UEBMI), and 1.6% (*N* = 172) did not have any medical insurance.

**Table 1:** Descriptive characteristics of participants.

**Variables**		**Frequency**	**Percentage (%)**
Gender	Male	4,846	45.7
	Female	5,754	54.3
Age (Years)	<30	6,119	57.7
	30–39	3,214	30.3
	40–49	818	7.7
	50–59	370	3.5
	>60	80	0.8
Monthly income (×1,000 RMB)	<3	2,678	25.3
	3–5	2,688	25.4
	5–10	3,902	36.8
	10–30	1,219	11.5
	>30	113	1.0
Education level	Junior high and below	245	2.3
	High school	1,439	13.6
	University degree	7,867	74.2
	Master degree and above	1,049	9.9
Occupation	Government related departments	582	5.5
	Employees of enterprises and public institutions	7,013	66.1
	Peasant	507	4.8
	Students	2,843	26.8
	Retirees	136	1.3
	Unemployed and laid-off workers	151	1.4
	Others	488	4.6
Marital status	Unmarried	5,334	50.3
	Married	5,168	48.8
	Divorced	86	0.8
	Widowed	12	0.1
Household registration	Urban	5,627	53.1
	Rural	4,973	46.9
Medical insurance	NRCMS	3,862	36.4
	URBMI	4,212	39.7
	UEBMI	3,842	36.2
	Commercial insurance	1,292	12.2
	No insurance	172	1.6

**Figure 1:**
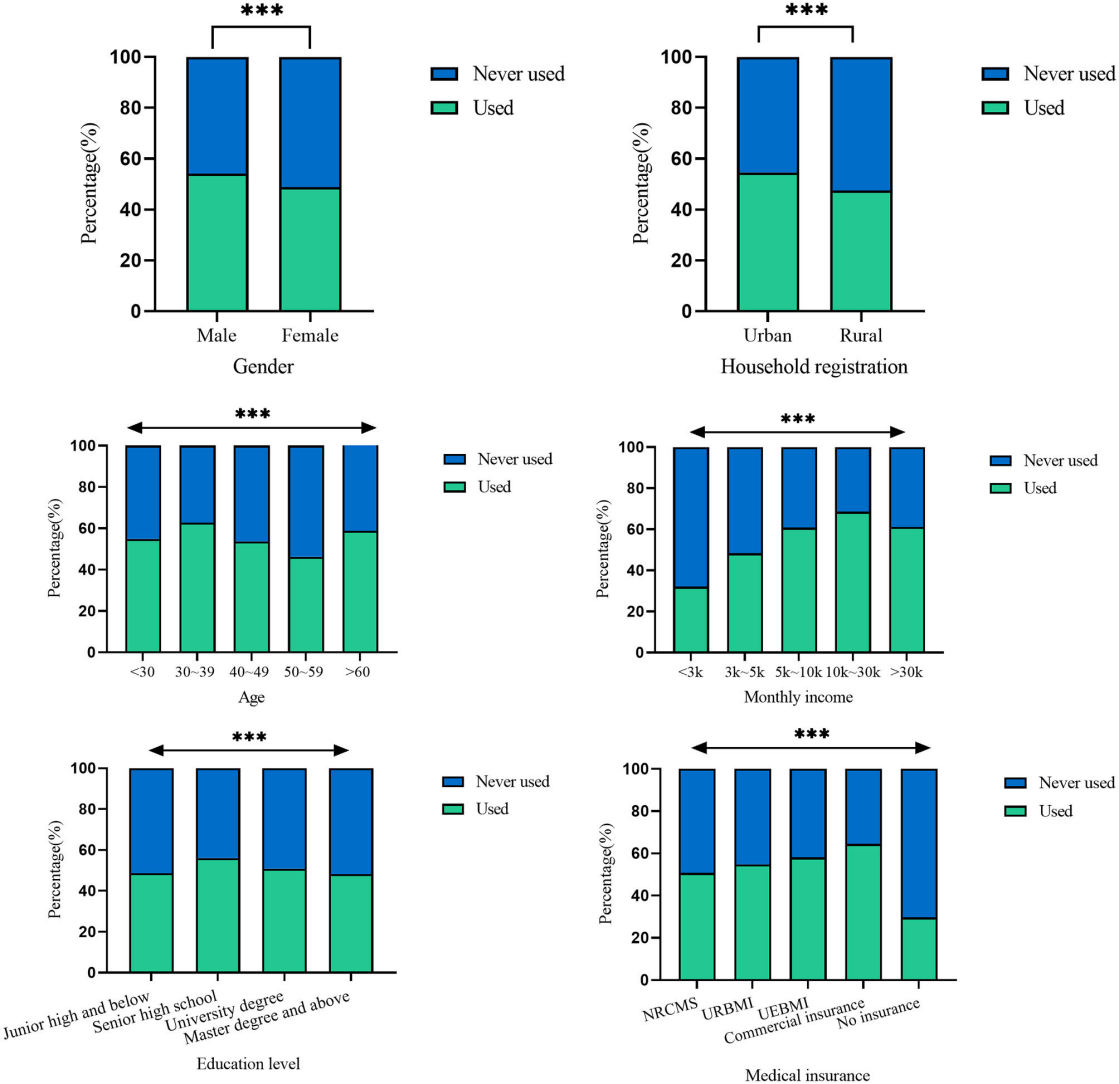
Use of Internet-based healthcare services provided by primary health institutions. ***p-value < 0.001.

Among participants, 51.3% (5,434) ever used Internet-based healthcare services provided by primary health institutions. Male individuals (54.1vs. 48.9% of female individuals) and those who lived in urban areas (54.6 vs. 47.5% of rural residents) were more likely to use the Internet-based services provided by primary health institutions than their counterparts (*P* < 0.05) (Figure 1). The respondents with commercial insurance had the highest utilization rate (64.5%) of the services, followed by UEBMI (58.1%), URBMI (54.8%), and NRCMS (50.8%). The respondents without any medical insurance had the lowest utilization rate (29.7%).

**Figure 2:**
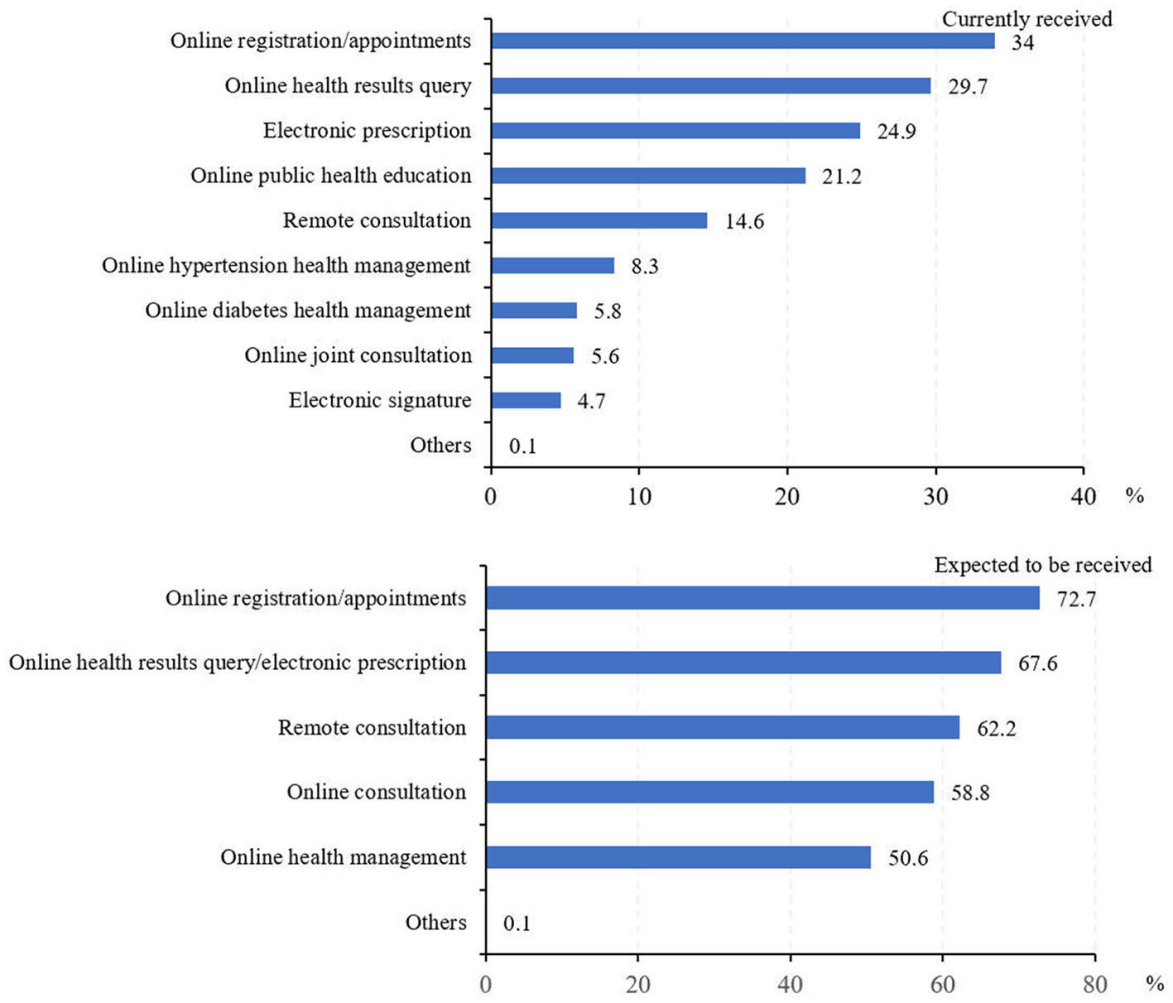
Types of Internet-based primary healthcare services currently received and expected to be received (%).

Figure 2 shows the types of Internet-based healthcare services provided by primary health institutions that people used and expect to use in the future. Overall, 34.0% of respondents used Internet-based services for online registration and medical appointment and 29.7% for online health results query, followed by online consultation (24.9%), online public health education (21.2%), and remote medical consultation (14.6%). The respondents who used Internet-based services for hypertension and diabetes health management accounted for 8.3 and 5.8%, respectively. Only 5.6% of the respondents used Internet-based joint consultation. By contrast, most people hoped to use online registration and appointment services (72.7%), followed by online electronic prescription and results query (67.6%), and remote consultation (62.2%). More than half of the respondents hoped to apply it in online consultation (58.8%) and online health management (50.6%).

**Figure 3:**
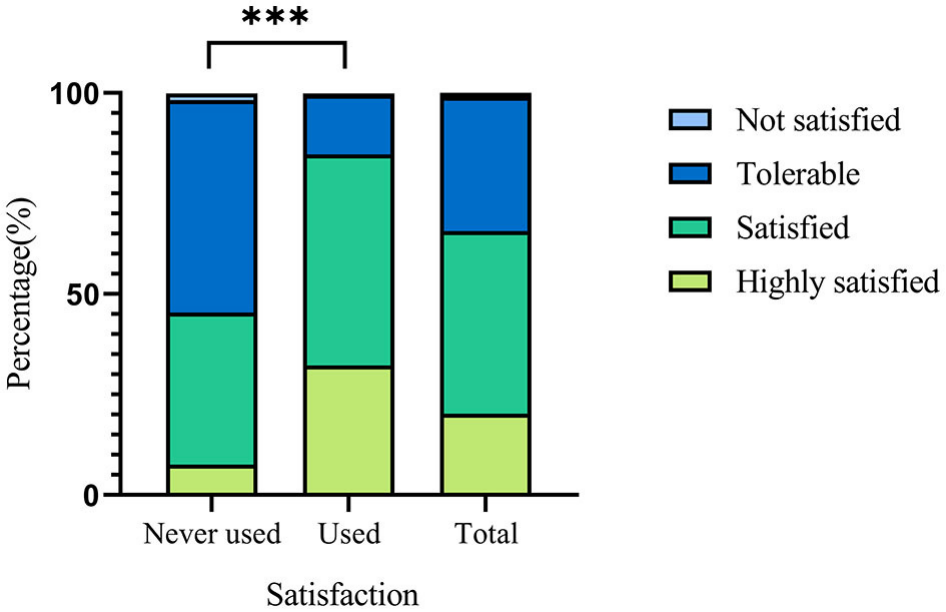
Satisfaction with Internet-based healthcare services provided by primary health institutions among participants who ever used and did not. ***p-value < 0.001.

**Figure 4:**
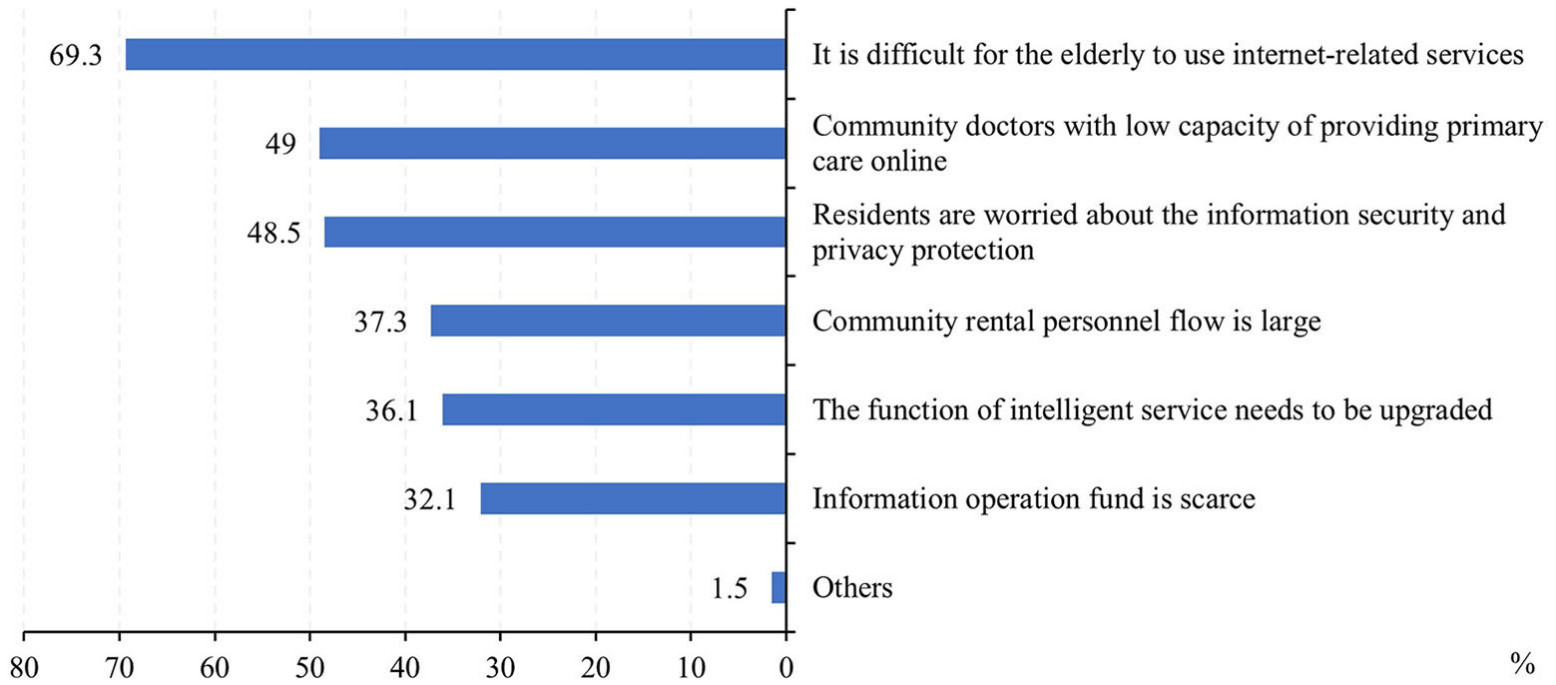
Reported challenges faced by Internet-based healthcare services in primary health institutions.

Figure 3 shows the satisfaction of people who ever used and those who did not use but knew about Internet-based primary healthcare services. It showed that those who ever used it had higher satisfaction (very satisfied or satisfied) rate than those who did not (84.7 vs. 45.4%). As for the challenges faced by the Internet-based healthcare services provided by primary health institutions (Figure 4), most people (69.3%) thought it was difficult to use Internet-based healthcare services for the elderly. More than half of the respondents considered that community doctors had a low capacity of providing primary care online (49.0%) and residents were worried about information security and privacy protection (48.5%).

Multivariable logistic regression (Table 2) showed that having chronic diseases, self-rating of health status, gender, and household registration was significantly associated with the utilization of Internet-based healthcare services (*P* < 0.05). People living in urban areas were more likely to use the Internet-based healthcare services provided by primary health institutions with OR = 1.095 (95% CI, 1.008–1.190). People with more than one chronic disease (OR = 1.704; 95% CI, 1.561–1.861) and higher self-rated health status (OR = 1.215; 95% CI, 1.179–1.252) were more likely to use it, while women were less likely to use it (OR = 0.870; 95% CI, 0.084–0.942).

**Table 2:** Association of risk factors of Internet-based healthcare service usage provided by primary health institutions from multivariable logistic regression model.

**Variables**	***P*-value**	**OR^a^**	**95% CIs**	
			**Lower**	**Upper**
Health status score	0.000	1.215	1.179	1.252
**Gender (Reference: Male)**				
Female	0.001	0.870	0.804	0.942
**Household (Reference: Rural)**				
Urban	0.031	1.095	1.008	1.190
**Whether have any chronic diseases or not (Reference: No)**				
Yes	0.000	1.704	1.561	1.861
**Whether have medical insurance or not (Reference: No)**				
Yes	0.150	0.795	0.582	1.086
**Age (Reference:**<**30)**				
30–39	0.000	1.736	1.584	1.904
40–49	0.378	1.068	0.923	1.235
50–59	0.830	1.028	0.796	1.328
>60	0.286	1.358	0.775	2.379
**Monthly income (**×**1,000 RMB, Reference:**<**3)**				
3–5	0.340	1.058	0.943	1.187
5–10	0.791	0.984	0.875	1.107
10–30	0.999	1.000	0.853	1.172
>30	0.210	0.776	0.522	1.153
**Education level (Reference: Junior high and below)**				
High school	0.174	0.822	0.620	1.090
University degree	0.106	0.801	0.612	1.048
Master degree and above	0.124	0.792	0.589	1.066
**Marital status (Reference: Unmarried)**				
Married	0.377	1.044	0.949	1.148
Divorced	0.416	1.202	0.772	1.872
Widowed	0.904	1.075	0.330	3.504

a OR, odds ratio.

## Discussion

### The utilization of Internet-based healthcare services

We found that 51.3% have used Internet-based healthcare services provided by primary healthcare institutions among 10,600 residents surveyed in China in 2022. However, most of the Internet-based healthcare used were procedure-related consultation services (63.7%), such as online registration, appointment booking, and result checking. One explanation for these findings may be that most medical institutions in China are now advocating and investing the information technology use within the hospitals (15). Relatively speaking, these services (i.e., online registration and appointment booking, and providing online result checking services) can take effect immediately after they are built, so they have become the priority developed by various medical institutions. In the context of China's large population and crowding in large hospitals, these online registrations and appointment booking services could better reduce the burden of seeking healthcare services among patients (16).

Online consults and teleconsultation were relatively less used in our study. In fact, with the building of a hierarchical medical system in China, the infrastructure for online consults, health management/interventions, and teleconsultation have been completed in most hospitals and primary health institutions, especially in the east of China (17). However, the utilization is particularly low. The reasons may include that in general, the distance between primary health institutions and large hospitals is relatively close, especially in the east of China, which causes the patients to choose to visit the large hospitals physically rather than using online or teleconsultation when they feel sick (18). In addition, subject to the limitations of telemedicine or online technology compared to face-to-face consultation and influenced by people's long-term medical habits, most people may believe that online diagnosis and treatment are not reliable to some extent (19). However, for people in remote rural areas with limited medical resources, Internet-based healthcare services could offer them access to high-quality and high-level medical services (20, 21). In addition, health management/interventions were also the services that people expect to be available in the future. Under the high prevalence of chronic diseases (e.g., diabetes, high blood pressure, and cancers), the related health behaviors and risk factors are increasingly a concern to populations with increasing health awareness (22). Moreover, health management and health risk factors intervention are one of the main objectives of primary healthcare institutions. Therefore, related Internet-based health promotion should be paid more attention in the future for healthy China construction.

### Satisfaction toward Internet-based healthcare services

People who have used the Internet-based healthcare services provided by primary health institutions were significantly more satisfied than those who have not used them. This may be because people have a bias against primary health institutions. The long-standing perception that primary health institutions have lower technical levels and lower service capability may affect their evaluation (23, 24). Therefore, it is necessary to publicize Internet-based healthcare services and to further promote the service capacity of primary health institutions.

### The influencing factors of utilization

The differences also existed in the utilization of Internet-based primary healthcare services among people with different characteristics. For example, people with medical insurance were more likely to use it than those without, which was consistent with the findings of previous research (25, 26). It is thus clear that medical insurance was still an important economic incentive to promote patients' access to healthcare. People with chronic diseases were more likely to use Internet-based healthcare services in primary health institutions, which was also consistent with the previous research (27). This may be related to the disease's nature and the necessity for follow-ups, and health-related Internet service is a convenient way to manage chronic diseases. In addition, people with higher self-rated health scores were more likely to use Internet-based healthcare services. One explanation may be that people who use the Internet for health purposes are more health-oriented than people who do not. However, the relationship between health status and the utilization of Internet-related healthcare remains controversial in published research partly subjective to the included research population and their concept of seeking medical services (28). In addition, as education level increases, people tended not to use Internet-based primary healthcare services. This finding is consistent with a previous study that found that a lower level of education is associated with higher utilization in primary healthcare (29, 30). This may be because people with a higher education degree apply higher standards in assessing the healthcare services received, so that even objectively better healthcare services do not meet their subjective standards of care, which may cause a relatively lower utilization.

### The challenges and suggestions

Regarding the challenges for the further development of Internet-based healthcare services, considering the difficulties regarding the use among the elderly, the services offered by primary health institutions should be further improved to meet the elderly's needs, which will also be urgent to adapt to the increasingly aging in China. Primary health institutions should increase investment in the age-appropriate renovation of Internet medical services, including the construction of hardware and software. Second, primary healthcare staff in China are usually inadequately trained and lack professional skills. The health sector should implement a system of continuing education, regular professional training, and competency tests for staff in primary health institutions. Third, when it comes to Internet-based healthcare services, China's legal system for protecting personal medical information and privacy is still in its infancy. Therefore, Chinese legislative, health, and information departments should join forces to develop sound laws and regulations for health information leakage.

### Strength and limitations

The findings of this study provide important insights into the use of, satisfaction toward, and challenges faced by Internet-based healthcare services of primary health institutions in China. Although there is much research on Internet-based healthcare in large hospitals, we focus on primary health institutions to further promote the achievement of universal healthcare. Moreover, the data were collected at the nationwide level, and participants from areas with different economic levels were incorporated. However, this study also has several limitations. First, as this was a cross-sectional study, meaningful differences could only be considered correlational, not causal. Second, the data were collected online, which may be subject to the limitation of online investigation. For example, users with multiple accounts may fill in the questionnaire repeatedly; the data might be, to a certain extent, affected by the quality of answers, although we set an “attention check” in the questionnaire to identify the careless respondents. In addition, it is possible that respondents who could not use smartphones were excluded from the study, which may lead to an overestimation of utilization. Third, considering China is a big country, the use of Internet-based healthcare services may vary greatly among people in different places of residence, so the sample of our study may not fully represent the specificity of different areas.

## Data availability statement

The raw data supporting the conclusions of this article will be made available by the authors, without undue reservation.

## Ethics statement

The study protocol and consent procedure were approved by Ethics Committee of School of Public Health, Zhejiang University School of Medicine (ZGL202203-6). The patients/participants provided their written informed consent to participate in this study.

## Author contributions

SW conducted the literature review, analyzed the data, and drafted the manuscript. JX contributed to the study conception and design and critical revisions of the manuscript. All authors were responsible for the structure of this study and approved the final versions for submission.
